# Monopolar Transurethral Enucleoresection of Prostate: Feasibility of Modified Nesbit’s Enucleoresection with Apical Release

**DOI:** 10.3390/jcm13051455

**Published:** 2024-03-02

**Authors:** Nitesh Kumar, Bhaskar Somani

**Affiliations:** 1 Ford Hospital and Research Centre, Patna 800027, India; niteshkal@gmail.com; 2 University Hospital Southampton NHS Foundation Trust, Southampton SO16 6YD, UK

**Keywords:** BPH, TURP, TUERP, MIST, prostate, laser

## Abstract

**Background**: Transurethral resection of the prostate (TURP) has been the standard surgical treatment for Benign Hyperplasia of the Prostate (BPH) for decades. Our objective was to evaluate the outcome of our new technique: Monopolar Transurethral Enucleoresection of the Prostate (TUERP) with apical release (bring it all to centre). **Methods**: A prospective study of all cases undergoing TUERP at a tertiary centre from January 2020 to October 2022 was performed. Patient demographics, intraoperative variables and postoperative results along with follow-up data were collected. Data of all the cases who had completed a one-year follow-up post-surgery were included and analysed. **Results**: A total of 240 patients with complete data including a one-year follow-up were included. Mean prostatic volume was 55.3 ± 11.6 gm, and 28 (11.67%) cases were >100 gm. The mean operative time was 31.7 ± 7.6, and mean haemoglobin drop at 24 h was 0.73 ± 1.21 gm/dL. The overall complication rate was 16.67%, with only two (0.83%) Clavien–Dindo III complications (haematuria and clots needing evacuation) and the other complications being Clavien–Dindo I/II complications. Sustained improvement at 1 year of follow-up was noted: Qmax: 25.2 ± 5.6 mL/s, IPSS: 4.7 ± 2.5 and PVR: 22.5 ± 9.6 mL. **Conclusions**: Monopolar TUERP with a modified Nesbit’s enucleoresection with apical release can be considered a promising technique, which needs further studies to be validated with appropriate comparisons.

## 1. Introduction and Objective

Transurethral resection of the prostate (TURP) has been the standard surgical treatment for Benign Hyperplasia of the Prostate (BPH) for decades. Even in the era of minimally invasive surgical therapies (MISTs), including laser enucleation, TURP remains the most commonly performed surgery for BPH [1]. The European Association of Urology (EAU) recommends monopolar TURP (M-TURP) or bipolar TURP (B-TURP) to surgically treat moderate-to-severe lower urinary tract symptoms (LUTS) in men with a prostate size of 30–80 mL [2]. Bipolar TURP achieves short-, mid- and long-term results comparable with M-TURP, but B-TURP has a more favourable peri-operative safety profile.

There have been concerns regarding complications related to the procedure, especially TUR syndrome, but its incidence is quite low (0.02%) [3]. The availability of modern-day optics and newer electrosurgical devices allow for very good resection, enucleation and haemostasis after the procedure, under full visual control. The morbidity related to M-TURP has decreased significantly over time due to these advancements, and the results are almost comparable with other techniques [4,5].

The morbidity, complications and inferior results of M-TURP is probably due to incomplete removal of the adenoma, multiple bleeders and absorption of fluid with poor apical resection. These drawbacks can be overcome during transurethral enucleation of the prostate (TUEP) even when performed with monopolar energy [6]. Enucleation of the prostate with laser technology (Holmium and Thulium lasers) are growing with time due to better haemostasis and complete anatomical removal of the adenoma with good long-term results [7].

The first description of M-TUEP was given by Hiraoka et al. [8] in 1986, where they used a detaching blade. However, enucleation techniques only became popular in the late 1990s with the introduction of Holmium laser enucleation of the prostate (HOLEP). While we perform HOLEP at our primary centre, due to the non-availability of lasers and morcellators at peripheral centres, we stuck with the mantra of “enucleation is enucleation is enucleation”, and that prostate adenoma should be removed in an anatomical manner regardless of the energy source [9]. Hence, we decided to start enucleation and simultaneous resection with the most easily available energy source and developed our monopolar TUERP technique: a modified Nesbit’s enucleoresection with apical release (bring it all to centre) for treating the prostate in a more anatomical manner. The technique preserves the sphincter, causes less blood loss and provides very good relief of obstruction without the need for postoperative traction. Our objective was to evaluate the outcome of this new technique.

## 2. Methods

A prospective observational study was conducted at Ford Hospital and Research Centre, a tertiary care centre, from January 2020 to October 2022. Ethical committee approval was obtained (FHRC/IEC/AUG-2020/002). All patients presenting with lower urinary tract symptoms (LUTS) suggestive of enlarged prostate were evaluated further. A detailed history, physical examination and basic preoperative blood and urine investigations were performed. International prostate symptom score (IPSS) was obtained, and digital rectal examination (DRE), uroflowmetry, serum prostate-specific antigen (PSA) and abdominal and transrectal ultrasonography (USG) for prostate volume were performed. Urodynamic study (UDS) was performed when indicated and as per clinician preference. The patients who were taken up for TURP after evaluation were included in the study.

Patients with a prostate less than 30 g (gm), serum PSA > 4 ng/mL, previous history of TURP, concomitant urethral strictures, and neurological diseases, those who were not willing to participate in the study and those who did not complete 12 months’ follow up were excluded from the study. Written and informed consent was taken from all patients. Active urinary tract infection (UTI) was treated before intervention. Anticoagulants were stopped 5 days before and started 5 days after or when haematuria subsided.

Spinal anaesthesia (SA) or epidural anaesthesia was administered for all patients, and they were kept in the lithotomy position. Otis urethrotomy was performed for all patients till 28F. A 26F Iglesias continuous irrigation resectoscope sheath (Karl Storz) with a monopolar working element and cutting loop was used for performing the surgery in glycine irrigation. An Allen combimax MBXVP model electro surgical generator was used for cutting (120 W) and coagulation (80 W).

**Figure 1:**
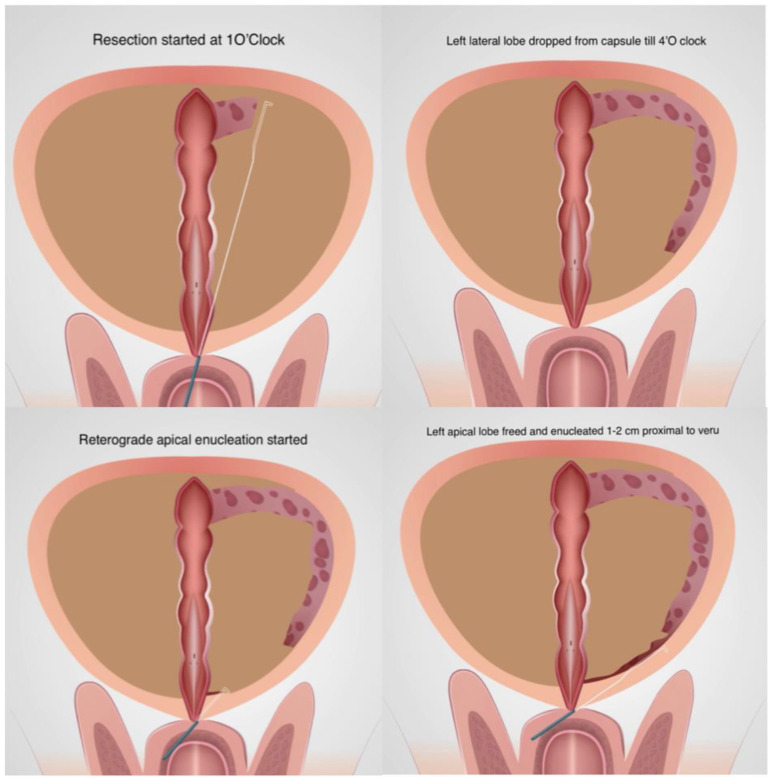
Enucleation of left lobe.

A diagnostic cystourethroscopy was performed first. Resection was started at the 1′O clock position and the left lateral lobe was dropped on the floor as in Nesbit’s technique. A plane of depth defined by capsular fibre was followed from the prostatic base to apex and the gland was detached from the capsule till 4′O clock. Careful haemostasis of all bleeders encountered was performed. The resectoscope was then brought to the apex of the prostate and the mucosa over the apical lobes were cut with the cutting loop and small bursts of monopolar cutting current. The apical tissue was then pushed mechanically towards the bladder to peel/enucleate it from the capsule for a distance of 1 cm proximal to verumontanum (Figure 1).

**Figure 2:**
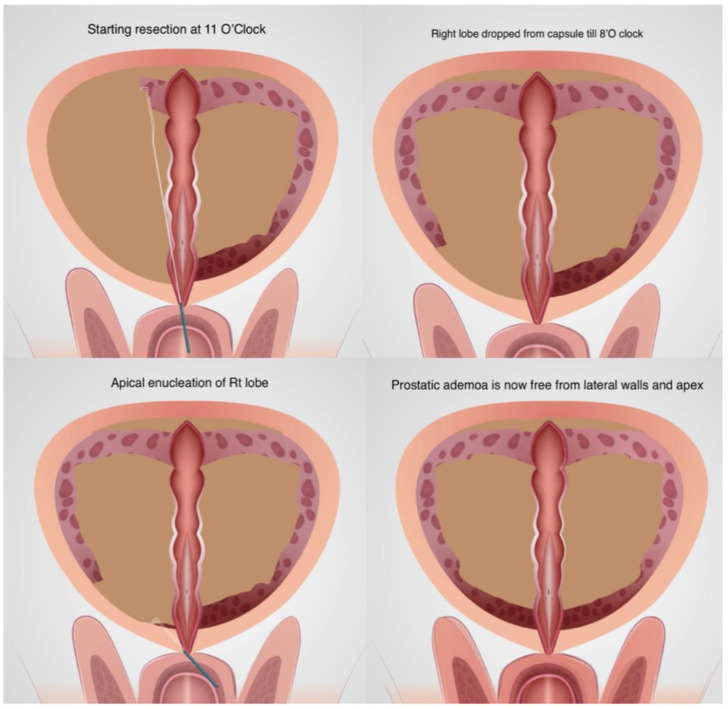
Enucleation of right lobe.

After leaving a strip of tissue and mucosa at 12′O clock, the right lobe resection was started from 11′O clock. Similar steps were repeated on the right side to free the prostatic tissue from both the lateral walls till 8′O clock and apex (Figure 2). The mucosa at the apex becomes free at the white line described by Gomez Sancha [10] and there is no traction to the sphincter.

**Figure 3:**
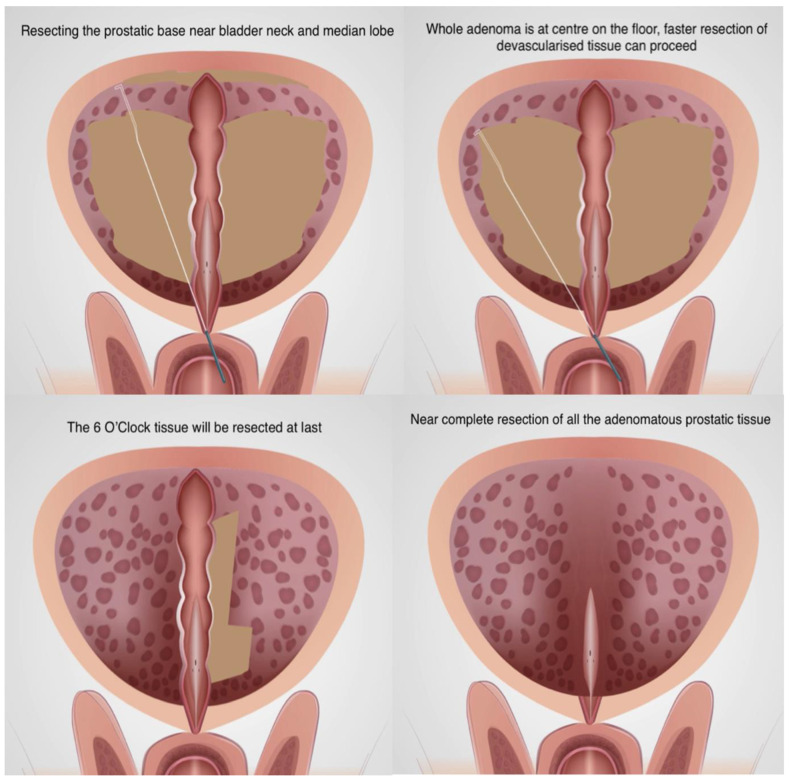
Resection at the bladder base to bring all tissues to centre on the floor.

The resectoscope was then brought to the bladder neck/prostatic base and the median lobe (if present) was resected along with the prostatic tissue till bladder neck fibres were visible. A careful haemostasis at the bladder neck was performed. Then, the whole bulk of the prostatic tissue was in the centre on the floor, free from the lateral walls, apex and base, with the only attachments at the 6′O clock mucosal strip and verumontanum. The gland became almost devascularised, and the remaining tissue bulk was resected at much faster speed (Figure 3).

Tissue chips were evacuated with Ellick’s evacuator and careful haemostasis was performed. A 20F 3-way foley catheter was placed and normal saline irrigation was started. If urine was haematuria, traction was applied for 2 min and then released when it cleared. If the urine continued to be haematuria, a re-look cystoscopy and careful coagulation of the bleeders was performed. Similar steps were repeated after placing the catheter and it was left without traction.

Patient demographics, intraoperative parameters and postoperative outcomes were recorded. Intraoperative and immediate postoperative complications were recorded and classified according to the Clavien–Dindo system. Patients were followed up at 3, 6 and 12 months, and IPSS, maximum urine flow (Qmax), postvoid residual urine (PVR) and prostate volume were recorded. Descriptive statistics were used, and data were analysed by XlStat 2021 software.

## 3. Results

We enrolled 250 patients in our prospective study of TUERP. Overall, 10 patients were excluded from analysis as they were lost to follow-up and the data of 240 patients were ultimately analysed.

The demographic variables of the study population are described in Table 1. Mean age was 62.7 ± 4.7 (range: 50–84) years and BMI was 23.7 ± 2.7 (range: 17–31). The mean prostatic volume was 55.3 ± 11.6 (range: 34–180) grams, of which 28 patients (11.6%) had prostates weighing more than 100 g. Mean IPSS score was 21.1 ± 4.7 (range: 9–34), mean Qmax was 11.4 ± 8.9 (range: 3–16) and mean PVR was 62.6 ± 21.1 (range: 28–400).

The intraoperative variables and the postoperative outcomes are described in Table 2 with a separate column for the prostates larger than 100 g. Mean operative time was 31.7 ± 7.6 (range: 15–90) min and mean weight of resected tissue was 43.4 ± 12.3 (range: 22–160) g, amounting to a resection efficiency of 1.36 gm/s which was increased to 1.80 gm/s in larger prostates. Mean haemoglobin (Hb) drop at 24 h was 0.73 ± 1.21 (range: 0.5–2.1) gm/dL and blood transfusion was required only in two (0.83%) patients.

Overall, 14 (5.83%) patients needed some attention to the catheter, which included traction in 3 (1.25%) patients, catheter change in 6 (2.5%), clot evacuation in 2 (0.83%) and flushing only in 3 (1.25%) patients. Mean catheter duration was 38.4 ± 11.6 (range: 24–96) h, 13 (5.41%) patients had haematuria >24 h, mean fall in sodium was 3.1 ± 0.5 (1.3–5.6) mEq/L and the residual prostatic weight at 1 year was 12.8 ± 2.6 (range: 8–25) g. There was not much difference between the overall data and the sub analysis of larger (>100 gm) prostates, except a slightly increased drop in haemoglobin and relatively increased resection efficiency.

Postoperative complications have been described in Table 3 Clavien–Dindo III complication was seen in two patients, who were re-transferred to the operation room for clot evacuation (one was done in local anaesthesia and the other one in spinal anaesthesia). All others (*n* = 38, 15.8%) were Clavien–Dindo I/II, which included transient stress urinary incontinence (SUI) in 6 (2.5%), need for blood transfusion in 2 (0.83%), 14 (5.83%) needing attention for catheter, prolonged irrigation in 13 (5.41%) and urinary tract infection (UTI) and dysuria in 3 (1.25%). Two (0.83%) patients developed urethral stricture, one at the submeatal level and another at the bulbar urethra. None of the patients had persistent SUI at 3 months, and none developed bladder neck contracture at 1-year follow-up. No TUR syndrome was noted in our series.

There was good reduction in IPSS score after surgery from 21.1 ± 4.7 (range: 9–34) to 7.2 ± 3.2 (range: 3–17) at 1 month, and there was persistent improvement of 5.4 ± 2.9 (range: 2–15) at 6 months and 4.7 ± 2.5 (range: 2–12) at 1 year. There was good improvement in Qmax from pre-treatment values of 11.4 ± 8.9 (range: 3–16) to 29.2 ± 6.1 (range: 22–50) at 1 month, which was sustained at 6 and 12 months of follow-up. PVR reduction was also achieved and improved at follow-up till 12 months (Table 4).

## 4. Discussion

The surgical treatment of BPH started from enucleation using an open method and then shifted to resection (TURP), but with the advent of lasers and enucleation techniques, there has been a paradigm shift towards enucleation [11]. TURP still holds the top spot amongst the different surgical treatment of BPH in the present era due to its easy learning curve, worldwide availability of equipment and low initial setup cost [11]. Different guidelines mention other techniques as an alternative to TURP for the treatment of BPH [1,2,12,13].

Since the first description of transurethral enucleation of the prostate by Hiraoka in 1986, who performed it with monopolar energy, there has been reconsideration of the old open enucleation technique [8,10,13]. Within a few years, lasers, bipolar energy and morcellators entered the market and endoscopic enucleation became an established technique for BPH, especially for glands >80 gms [14,15,16]. But the availability, cost and a long steep learning curve became a barrier in its universal adoption [11,12]. It is likely that most surgeons would be ready for the long learning curve, especially when the new technique is a modification of an old established TURP technique, with a fail-safe method known to them.

The enucleated lobes at the bladder neck can make it difficult to resect, as the tissue is unstable and becomes more troublesome when lobes/large tissue chunks fall into the bladder [17]. Therefore, we decided against complete retrograde enucleation and devised a novel modification which brings all the prostatic tissues to the centre for a fast and rapid resection. The technique starts with Nesbit’s method of dropping the lateral lobes and then proceeds to apical release and enucleation up to the mid-prostate, followed by resection at the bladder neck. It devascularises the whole gland and brings all the tissues to the floor, at the centre, allowing for a rapid resection of larger glands within the permissible time limit for monopolar energy and glycine irrigation. The most difficult areas for enucleation (monopolar/bipolar) are the roof and bladder neck, and both these areas are dealt with using the more familiar transurethral resection with the loop, decreasing the learning curve.

Pansadoro [18] described his monopolar enucleation technique (47 cases) in 2017, where he divided the prostate into three lobes, median, left and right, by making a groove with an angled loop and then enucleated them with a straight loop, which were then removed by a morcellator. His mean prostatic volume was 64.9 g, slightly more than our study, but his mean operative time was 126.41 min (resection efficiency: 0.31 gm/min), which was four times greater than in our study (31.7 min). He reported complications in 27.6% patients (16.6% in our study), out of which 7% comprised Clavien–Dindo class III. Surprisingly, TUR syndrome was reported in none of the patients, even with such operative times, emphasizing the fact that there was far less fluid absorption if enucleation was performed.

Enikeev [19], in his comparative study of 551 patients (with glands >80 g), performed mTUEP on 95 patients. They used a hook electrode to incise the floor at 5′o clock and the roof at 12′o clock from the bladder neck to the verumontanum and then connected them at the apex of the prostate. The lobes were then enucleated with the help of a straight loop and morcellated. They reported similar postoperative outcomes and complication rates for HOLEP, ThuLEP and mTUEP, but the operative time, fall in hemoglobin and sodium was significantly higher in the mTUEP group. The resection efficiency was 0.81 g/min in mTUEP and 0.97 g/min in HOLEP, which was reduced compared to our study (1.36 g/min). An interesting result in his study was that no urethral orifice damage was encountered in mTUEP compared to two cases and one case in HOLEP and ThuLEP, respectively, establishing the safety of mTUEP.

Enikeev [6] compared monopolar enucleation (70 cases) with TURP (64 cases) in glands <80 g again in 2020. The mean prostatic volume was around 60 g and resection efficiency was 1 gm/min in both the resection and enucleation groups, but the catheterization time, hospital stay and residual prostate volume was significantly less in the enucleation group. The complications and short-term results in terms of IPSS, Qmax and PVR were similar. They reported complications like TUR syndrome and excessive bleeding with larger prostates, which were not issues in the studies of Ajib [20] and Pansadoro [18]. They found that the learning curve of monopolar enucleation was slightly higher than HOLEP but concluded that monopolar enucleation is a relatively safer procedure as no serious complications were encountered during the early learning curve.

Ajib [20] described the TUERP technique in comparison with HOLEP in 2019 (24 vs. 27 cases); they resected the median lobe in a similar manner to TURP and then enucleated the lateral lobes. If the lobes were completely enucleated, they was morcellated or else resected with a loop. The mean prostatic volume was 87.2 g and the resection efficiency was 0.47 gm/min. The short-term postoperative results and complications were comparable between the two groups. The most difficult part at 12′o clock was enucleated at last. There was no predefined technique for enucleation and removal of the adenoma. We have described the enucleation and resection part step by step and used a single consistent technique for this.

Li K [21] compared M-TUERP (42 cases) with B-TURP (44 cases) in 2018 in prostates measuring >80 g. He used a suprapubic trocar in an attempt to reduce the bladder pressure and concluded that it resulted in a reduction in bleeding, clear field of vision and less hyponatremia. He resected the anterior fibromuscular stroma first from 10 to 2′O clock till the capsule was visible and then incised the tissues beside the verumontanum to reach the plane of enucleation at the apex. It was then connected with the previous resected plane and the gland was enucleated. He also left a small strip attached at 6′O clock to prevent tissues from falling into the bladder. His resection efficiency was 0.71 gm/min and reported significantly reduced catheter time and blood loss, residual gland and complications in the M-TUERP group; other short-term results and complications were similar.

Geavlete B [22] first described the bipolar enucleation in prostates >80 g based on similar principles of HOLEP. Liu [16], Wei [17], Palaniappan [23] and Zuo [24] described the TUERP with bipolar technique in a similar manner. True bipolar technology comes with a cost and is not available universally, and there are potentially more rates of urethral strictures with quasi-bipolar technologies [25]. We have replicated the same technique with bipolar TURIS and it can be performed with ease and similar efficacy.

Our technique is unique due to the fact that we preserved a strip of tissue with mucosa at 12′O clock, which helps in early epithelization, reducing bladder neck contracture and decreasing SUI. Another uniqueness of our technique is the resection of tissues at the bladder neck after the lobes are enucleated, up to the mid-prostate on the floor. It significantly reduces the learning curve, as the most difficult part of enucleation starts after the mid-prostate, from where it takes an upward curve. Capsular perforation and trigonal flaps with ureteric injuries can occur during this part of surgery, which is avoided by resection of the bladder neck. The third advantage which our technique offers is a very rapid and avascular resection after all the tissues have been brought to the centre, thus increasing the resection efficiency. In our subset of patients >100 g, both the enucleation and resection efficiency increased significantly.

We could not add a comparison arm in our study, but we compared our data with the available literature. The primary outcome: improvement in Qmax at 6 and 12 months was 27.5 ± 5.8 and 25.2 ± 5.6 in our study. A large meta-analysis (MA) by Bruce et al. [26] reported 17.87 ± 4.93 and 16.57 ± 1.03 in M-TURP and 18.60 ± 3.27 and 17.65 ± 1.41 in B-TURP at 6- and 12-month follow up. IPSS scores at 6 and 12 months were 5.4 ± 2.9 and 4.7 ± 2.5 in our study, compared to 9.25 ± 1.94 in the M-TURP and 8.35 ± 0.36 in the B-TURP groups in the above MA. This value was 6.69 ± 1.94 in the MA by Cornu et al. [27].

Some secondary outcomes were also compared with MA by Bruce et al. [26]. Our mean duration of catheterization was 38.4 ± 11.6 h compared to 57.3 ± 19.8 and 45.0 ± 18.5 h for monopolar and bipolar TURP, respectively in MA. A total of 5.8% needed catheter attention for clot retention in our study compared to 5.2% and 5.3% in M-TURP and B-TURP groups in the MA. The need for blood transfusion was comparatively lower in our study at 0.83%, vs. 11.3 and 6.7% in M-TURP and B-TURP groups. Acute urinary retention post-surgery for M-TURP and B-TURP was 4.2 and 4.4%, respectively, and TUR syndrome was found in 5.8% in the MA, but none was reported in our study.

The percentage of tissue removed was 78.3% by our modified technique. Rosenhammer et al. [28] reported 63.5% and 49.5% tissue removal in their matched pair comparative analysis of HoLEP and bipolar TURP. PVR at 12 months was 22.5 ± 9.6 mL in our study compared to 20.83 ± 9.81 in B-TURP and 25.14 ± 5.98 in M-TURP in the MA by Cornu et al. [27].

Complications of B-TURP and M-TURP were analysed in a large meta-analysis by Cornu et al. [27]. Haemoglobin loss at 24 h was 1.01 ± 0.43 and 1.81 ± 0.85 gm/dL in the MA for M-TURP and B-TURP, respectively, versus 0.73 ± 1.21 gm/dL in our study. Sodium decrease was 1.28 ± 0.49 and 3.47 ± 1.63 mol/L for M-TURP and B-TURP in the MA, versus 3.1 ± 0.5 mol/L in our study. Urethral stricture at 12 months was 3.2% and 3.1% for M-TURP and B-TURP in the MA, compared to 0.83% in our study. Blood transfusion rates were 2.63% and 5.5% for M-TURP and B-TURP, respectively, in the MA, compared to 0.83% in our study. Incontinence rates at 1 year were 1.5% and 2.2% in the MA for M-TURP and B-TURP, respectively, but none of our patients were incontinent beyond 90 days. Reoperation rates were 6.5% and 7.3% at 1 year in MA for M-TURP and B-TURP, respectively, and 0.83% in our study.

Sinha et al. [29] reviewed 18 randomized control trials which compared M-TURP vs. B-TURP with a total of 8393 patients. They reported a significant fall in haematocrit and greater occurrence of TUR syndrome in the M-TURP group, but we overcame these demerits of M-TURP by modified enucleoresection. The cut-and-seal principle of B-TURP, which led to less blood loss and less transfusion, was achieved with our technique where the gland was detached from the capsule and the bleeders were immediately sealed, leaving it devascularised. The proper assessment of gland and capsular depth, reduced opening of sinuses, immediate control of bleeders and faster resection led to reduced occurrence of TUR syndrome and we also completed larger gland enucleoresections without a single incident of TUR syndrome. Morozov et al. [30] performed an MA and compared the long-term outcomes of endoscopic enucleation (EEP) of the prostate and TURP. They found better long term Qmax, IPSS, PVR and PSA reduction in EEP. Our technique could achieve comparable results with that of EEP, with similar outcomes.

There is a need to evolve the enucleation technique for the treatment of BPH described by Gómez-Sancha [31] regardless of the available energy source [9]. It is interesting to note that most of the M-TUERP and B-TUERP techniques were described after HOLEP became established, and were established by surgeons who could perform HOLEP. The knowledge and experience of finding the enucleation plane led to the modification from resection to enucleation. It was driven by the superior results of enucleation techniques and the lack of lasers at all centres. We also modified the technique, initially starting with small glands, but with experience we were able to operate up to 180 g in 90 min.

The M-TUERP is a safe procedure with superior results, less bleeding, better resection efficiency, more complete adenoma removal, better and sustained reduction in PVR and IPSS scores and better and sustained improvements in Qmax. There are concerns about sphincter traction and greater SUI in the postoperative period due to mechanical enucleation. Our study showed SUI lasting >7 days in 2.5% cases and >30 days in 1.25% cases, but continence was achieved in all patients at 90 days. Our study was limited by the lack of a comparative arm, but a prospective comparative study with bipolar enucleoresection and HOLEP is ongoing and will be completed by the end of 2024. Similarly, it might be worth comparing outcomes with the newer minimally invasive surgical therapies (MIST) [32,33], especially in relation with not just surgical outcomes, but also the quality of life of these patients.

## 5. Conclusions

Monopolar TUERP with a modified Nesbit’s enucleoresection with apical release (bring it all to the centre) can be considered a promising technique, which needs further studies to be validated with appropriate comparisons. It preserves the sphincter, helps in faster resection, achieves near-complete removal of the adenoma, causes less blood loss, needs less irrigation and obviates the need for postoperative traction with very good results in terms of postoperative clinical outcomes.

## Figures and Tables

**Table 1:** Demographic parameters of the study population.

	Parameters	Results	Median (IQR)
1	No of patients	240	
2	Mean age (years)	62.7 ± 4.7 (50–84)	61 (56.75–65)
3	BMI	23.7 ± 2.7 (17–31)	23 (21–26)
4	Mean prostate volume (cm^3^)	55.3 ± 11.6 (34–180)	45 (39–56)
5	Prostate more than 100 gm	28 (11.67%)	
6	Mean prostate vol (>100 gm)	132.65 ± 20.35 (105–180)	129.5 (116.5–147.25)
7	PSA (ng/dL)	2.1 ± 0.8 (0.6–4.0)	2.3 (0.75–3.3)
8	No of patients requiring urodynamic study	15 (6.25%)	
9	No of patients on aspirin	71 (29.5%)	
10	Comorbidities (0:1:2:>2)	56(23.3%)/64(26.7%)/82(34.2%)/38(15.8%)	
11	Duration of symptoms (months)	15.2 ± 4.8 (8–31)	13 (10–19)
12	IPSS Score	21.1 ± 4.7 (9–34)	22.5 (15–26)
13	Q_max_ ( mL/s)	11.4 ± 8.9 (3–16)	12 (6–15)
14	Postvoid urine residue (mL)	62.6 ± 21.1 (28–400)	47 (37.25–65)
15	No of patients on catheter	31 (12.92%)	

**Table 2:** Intraoperative variables and postoperative outcomes.

	Parameters	Results	Prostates > 100 gm
1	Mean operative time (minutes) Median (IQR)	31.7 ± 7.6 (15–90), 23 (18–41.5)	65.3 ± 11.1 (50–90), 63.5(56.2)
2	Weight of resected tissue (grams) Median (IQR)	43.4 ± 12.3 (22–160), 31 (25–45.25)	117.6 ± 18.2 (91–160), 114.5 (103.7–128.7)
3	Resection efficiency (g/min)	1.36	1.80
4	Mean amount or irrigation used (litre) Median (IQR)	14.3 ± 3.1 (7–24), 14 (10.75–17.25)	17.7 ± 2.4 (15–24), 17 (15.25–19)
5	Duration of postoperative irrigation Median (IQR)	20.1 ± 2.7 (6–44), 20.5 (14.25–29.25)	20.8 ± 3.1 (12–42), 20 (14.5–26.5)
6	Mean haemoglobin drop @24 h (gm/dL) Median (IQR)	0.73 ± 1.21 (0.5–2.1), 0.6 (0.5–0.8)	1.17 ± 1.13 (0.8–2.3) 1.1 (1–1.2)
7	Catheter needing attention	14 (5.83%), Traction 3 (1.25%)	2 (7.1%), Traction: 0
8	Catheter change and clot evacuation	6 (2.5%), clot evacuation: 1 LA, 1 SA	1 (3.5%), clot evacuation: 0
9	Duration of catheter (hours) Median (IQR)	38.4 ± 11.6 (24–96), 32 (26–41.5)	41.3 ± 12.1 (30–84), 38 (36–43.5)
10	Postoperative haematuria >24 h	13 (5.41%)	2 (7.1%)
11	Blood transfusion	2 (0.83%)	0 (0%)
12	Residual prostatic volume at 1 year Median (IQR)	12.8 ± 2.6 (8–25), 12 (9–15.75)	15.4 ± 2.9 (12–22), 14 (13–18.75)
13	Fall in sodium at 24 h Median (IQR)	3.1 ± 0.5 (1.3–5.6), 3.3 (1.9–4.1)	3.6 ± 0.7 (1.8–5.4), 3.6 (2.9–4.5)

**Table 3:** Postoperative complications.

Sl No	Parameters	Results	Results (>100 gm)
1	Complications (Clavien–Dindo)	I/II—38 (15.8%), III—2 (0.83%)	I/II—4 (14.3%), III—0 (0%)
2	Stress urinary incontinence (SUI)	>7 days: 6 (2.5%), >30 days:3 (1.25%), >90 days: 0 (0%)	>7 days: 1 (3.5%), >30 days: 1 (3.5%), >90 days: 0 (0%)
3	Urethral stricture	2 (0.83%)	0 (0%)
4	Bladder neck contracture at 1 year	0 (0%)	0 (0%)

**Table 4:** Results of a short-term follow-up.

Parameters	Presentations	1 Month	6 Months	1 Year
IPSS	21.1 ± 4.7 (9–34)	7.2 ± 3.2 (3–17)	5.4 ± 2.9 (2–15)	4.7 ± 2.5 (2–12)
Qmax	11.4 ± 8.9 (3–16)	29.2 ± 6.1 (22–50)	27.5 ± 5.8 (20–50)	25.2 ± 5.6 (12–50)
PVR (mL)	62.6 ± 21.1 (28–400)	31.1 ± 15.6 (5–65)	26.8 ± 12.9 (0–52)	22.5 ± 9.6 (0–45)

## Data Availability

Data are contained within the article.
